# Immune-driven serotype-specific evolution of dengue virus NS1: Insights from structural and epitope analyses of Indian isolates

**DOI:** 10.1016/j.meegid.2026.106021

**Published:** 2026-11

**Authors:** Khushi Khera, Sanjeev Kumar, Anmol Chandele, Amit Sharma

**Affiliations:** International Centre for Genetic Engineering and Biotechnology, 110067 New Delhi, India

**Keywords:** Dengue virus, Non-structural protein 1 (NS1), Haplotype analysis, Selection pressure, Diversifying selection, B-cell epitopes, Immune exposure, Structural mapping

## Abstract

**Objectives:**

Despite the diagnostic and immunological importance of NS1, how evolutionary forces shape NS1 evolution among different dengue serotypes remains poorly understood.

**Methods:**

NS1 sequences representing all four dengue virus serotypes were analyzed for haplotype diversity and selection pressure using codon-based models. Sites showing evidence of diversifying selection were structurally mapped onto dimeric, tetrameric and hexameric NS1 conformations. Diversifying sites were also overlaid with predicted linear B-cell epitopes. Structural mapping of selected sites was done on NS1-antibody structure. Minimum heavy-atom distance of those sites to the antibody binding interface and predicted affinity change (ΔΔG) of each substitution were calculated.

**Results:**

Haplotype frequency analysis showed high haplotype richness and multiple co-circulating variants in DENV-2, moderate haplotype diversity in DENV-1 and 3, whereas DENV-4 showed lowest observed haplotype richness. Selection pressure analyses identified several sites showing evidence of diversifying selection, predominantly localized within the wing and β-ladder domains. These sites also overlapped with some conserved predicted linear B-cell epitopes. Notably, none of the analyzed diversifying sites directly overlapped with the binding interface of the antibody and were associated with minimal predicted changes in binding affinity.

**Discussion:**

Our results indicate that NS1 evolution reflects strong functional constraint with localized, serotype-specific variation, underscoring the relevance of continued NS1 sequence monitoring for diagnostic robustness. Conserved regions may warrant further investigation as candidate immunogenic regions.

## Highlights

• NS1 evolution across all four dengue serotypes was analyzed in Indian dataset.
• Positively selected sites were identified using multiple complementary codon-based models.
• Diversifying residues falls under surface exposed regions of wing and β-ladder domains of NS1.
• Several positively selected sites overlap with predicted B-cell epitopes and known immunodominant regions.
• Structural mapping reveals constrained evolution with minimum immune drive variation.

## Graphical abstract

**Figure:**
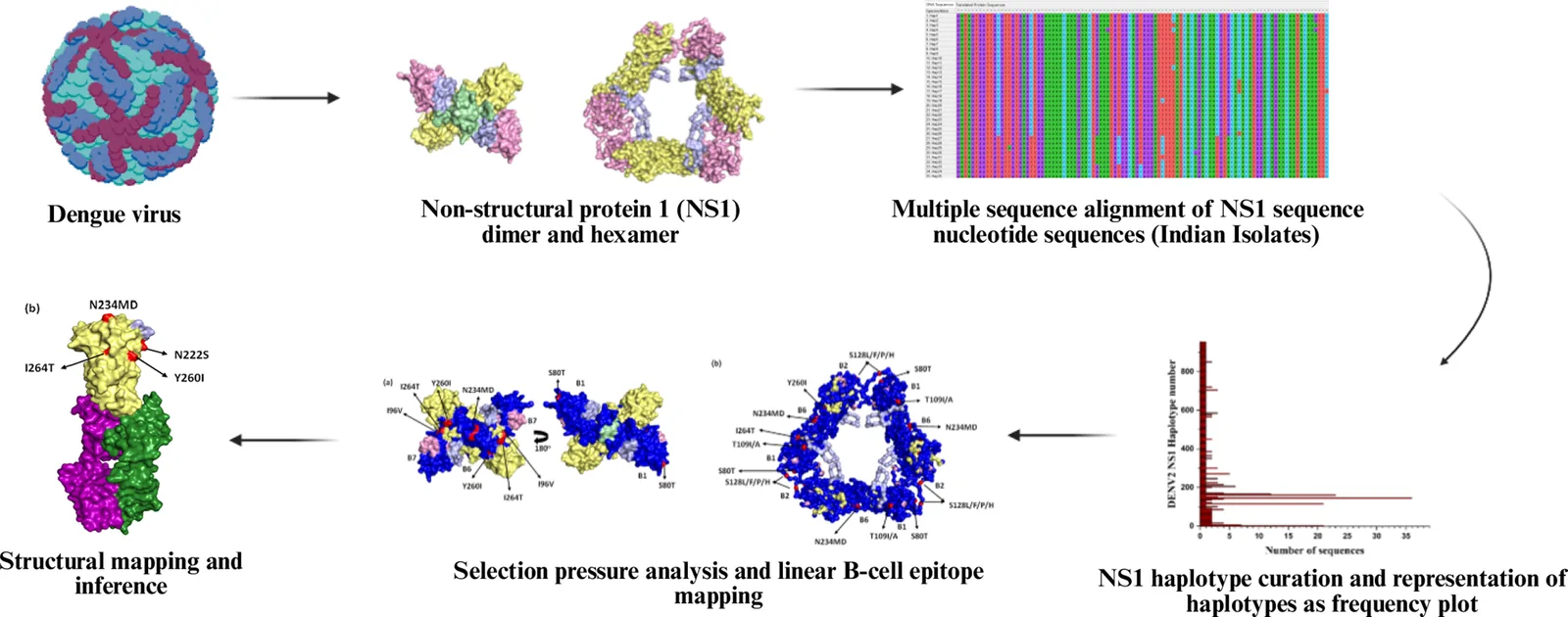

## 1 Introduction

Dengue virus (DENV) is one of the most prevalent arthropod-borne pathogens, which commonly spreads through the bite of *Aedes aegypti* and *Aedes albopictus* mosquitoes (Bhatt et al., 2021). Belonging to the *Flaviviridae*, genus Flavivirus, it is predominantly found in the tropics and subtropics like Southeast Asia and the American region. In the past few years, incidence of annual dengue cases has undergone a dramatic increase worldwide. According to the NCVBDC report, till April 30, 2026, there were 13,336 confirmed cases of dengue with 12 deaths across India (Dengue Situation in India, NCVBDC, 2026). DENV is classified into four antigenically distinct serotypes, DENV-1 to DENV-4. A sylvatic dengue virus strain was proposed as a potential fifth serotype in 2015, although this classification has not been incorporated into the conventional four-serotype framework (Bhatt et al., 2021; Mustafa et al., 2015). DENV infection is usually asymptomatic; however, in some cases, it can present a spectrum of clinical outcomes ranging from mild febrile illness to severe dengue. The 2009 WHO classification categorizes dengue into dengue without warning signs, dengue with warning signs, such as abdominal pain, persistent vomiting, mucosal bleeding, hepatomegaly, and hematocrit rise with thrombocytopenia and severe dengue (Nasir et al., 2024; Perera et al., 2024). India represents a hyperendemic setting for DENV, where all four serotypes co-circulate and sequential infections are common, creating sustained immune pressure on viral antigens. In North India, mainly in Delhi and Uttar Pradesh, DENV-1 and DENV-2 co-circulated in the past few years. DENV-2 was predominant until 2016, followed by the emergence of DENV-3 (Yadav et al., 2025). In South India, DENV-1 and DENV-2 were prevalent between 2011 and 2015, with a shift to DENV-4 (genotype I) in Karnataka, Tamil Nadu, and Puducherry after 2016. In eastern India, mainly in Odisha, DENV-2 was present during 2011–2013, followed by DENV-3. In Western India, especially Maharashtra and Rajasthan, DENV-3 was the predominant circulating serotype from 2014 to 2018, although DENV-2 and DENV-4 were also reported. In Central India, early reports indicated co-circulation of DENV-1 and DENV-4, but subsequent years showed predominance of DENV-1, DENV-2, and DENV-3, with scarce DENV-4 cases. In contrast, North-East India initially reported DENV-3 dominance in 2012, followed by a shift to DENV-1 in 2015 and later to DENV-2 (genotype IV) as the principal circulating serotype after 2016 (Yadav et al., 2025). Over the past decade, studies have reported the co-circulation of all four DENV serotypes within the same geographic and ecological settings (Sirisena et al., 2021). The co-circulation of multiple serotypes creates opportunities for concurrent infections, where individuals are infected with more than one serotype at a time. Despite a similar infection pathway, virological differences lead to diverse clinical manifestations across all the serotypes (Sirisena et al., 2021). Sequential infections with different DENV serotypes have also been reported (Phanthanawiboon et al., 2025; Reddy et al., 2017; Shivaswamy et al., 2022; Vinodkumar et al., 2013). Secondary infection of dengue with a heterologous serotype often result in more severe symptoms due to antibody-dependent enhancement (ADE) mechanism (Sarker et al., 2023). In ADE, the neutralizing effect of primary infection-induced serotype-specific antibodies provide a lasting immunity against the infecting serotype. However, during secondary infection, these cross-reactive (due to 60–70% sequence diversity between all serotypes) but sub-neutralizing antibodies can facilitate entry into Fcγ receptor-bearing cells such as macrophages, in turn, enhancing viral replication (Soo et al., 2016). Clinical studies have demonstrated that secondary infections are more frequently associated with severe outcomes, particularly when caused by DENV-2, DENV-3, or DENV-4 (Banu et al., 2025). Secondary infections with DENV-2 are commonly linked with severe bleeding and organ involvement, whereas DENV-3 is often associated with plasma leakage and respiratory distress (Banu et al., 2025).

Our focus here is the NS1 protein (Non-structural protein 1) of DENV which functions by binding to host cell-surface glycosaminoglycans (GAGs), attaching specifically to endothelial cells and platelets via the wing domain, particularly the GDI (G91, D92 and I93) motif and WWG (W115, W118 and G119) motif. This results in activation of platelets and hyperpermeability of endothelial cells (Kraivong et al., 2025, Lo et al., 2022). N130 glycosylation contributes to NS1 stability and its interactions with components of the complement system. Autoantibodies to NS1 regions which mimic host proteins result in cross-reactivity leading to heightened pathogenic responses. C-terminal β-ladder region (305–330) is known to cross react with proteins like human plasminogen, ATPase and protein disulfide isomerase (Chuang et al., 2016). NS1 exists as membrane-associated (mNS1) complex which localizes to vesicular and plasma membranes, and a secreted soluble form (sNS1) (Tan et al., 2023). The mNS1 is crucial for viral replication and colocalizes with replication complex components in both mammalian and insect cells. The sNS1 exists as a hexameric lipoprotein particle with open-barrel architecture and a lipid-rich core resembling high-density lipoproteins. Structural studies have also identified tetrameric assemblies under experimental conditions, likely representing transitional or intermediate conformations rather than stable circulating species (Alvin Chew et al., 2024; Shu et al., 2022). NS1 comprises three major structural domains: the β-roll (residues 1–29), wing (residues 30–180), and β-ladder (residues 181–352) domains. The wing domain contains an α/β subdomain (residues 38–151) and a discontinuous connector subdomain (residues 30–37 and 152–180) (Alvin Chew et al., 2024; Scaturro et al., 2015). The connector subdomain has a hydrophobic protrusion called the “greasy-finger” loop which has been implicated in membrane association and viral replication (Fig. 1, Supplementary Table S1) (Akey et al., 2014). The wing and β-ladder domains also contain glycosylation sites, N130 and N207 which are important for assembly of stable NS1 dimers (Fang et al., 2023). Additionally, the function of each structurally significant domain is summarized in Supplementary Table S1. To date, several epitopes have been identified on NS1 protein using many using *in vitro* approaches such as phage display, peptide arrays, and IgG and IgM ELISAs in mice and humans both in mice and in humans (Nagar et al., 2020). An illustrative and tabular summary of NS1 epitopes can be found in Supplementary Fig. S1, Supplementary Table S2. Additionally, Masrinoul et al. (2011) have reported amino acids 141–168 in NS1 which is highly conserved among all four serotypes and is recognized by mAb 2H11 and 3C4 (Masrinoul et al., 2011). A computational study by Valotto et al. suggests region 301–319 to be a candidate epitope for both dengue and Zika virus, recognized by mAbs 22NS1, 2B7 and 1G5.3, which can provide a specific target for diagnosis while circumventing cross-reactivity among other flaviviruses (Valotto et al., 2026). Beyond epitope level considerations, amino acid variations in NS1 can significantly influence viral transmission, particle secretion, and outbreak severity. For instance, the T164S substitution in NS1 was linked to the 1997 DENV-2 outbreak in Cuba (Rodriguez-Roche et al., 2011), while the G53D mutation in the DENV-2 16681 strain affects viral infection and dissemination in both mosquito and mammalian cells (Choy et al., 2020). Additionally, alanine substitutions at NS1 residues S114, W115, D180, and T301 were shown to alter the production of infectious virions, although viral RNA replication remained unaffected (Scaturro et al., 2015). Analysis of DENV-2 strains from the Southern Taiwan outbreak in 2015 revealed an NS1 K272R substitution, which promoted immune evasion by suppressing STAT1 phosphorylation and downstream ISG expression, while enhancing secretion of soluble NS1. This increase in sNS1 further activated the NFκB pathway and stimulated pro-inflammatory cytokine production compared to the wild-type virus, thus worsening the manifestation of the disease (Hee et al., 2025). In contrast, NS1 mutations such as V236A in DENV-2 or W68 (stop) in DENV-3 were associated with reduced NS1 secretion, as identified in NS1 ELISA-negative clinical isolates that were later confirmed to contain viral RNA (Ghosh et al., 2022). Additionally, *in vitro* studies have shown that mutations at N-glycosylation (N130A), phosphorylation (T29A, T230A, S233A), or residues like G53D can significantly reduce production of infectious virus without affecting intracellular NS1 levels or its secretion (Choy et al., 2020; Tajima et al., 2008).

**Fig. 1:**
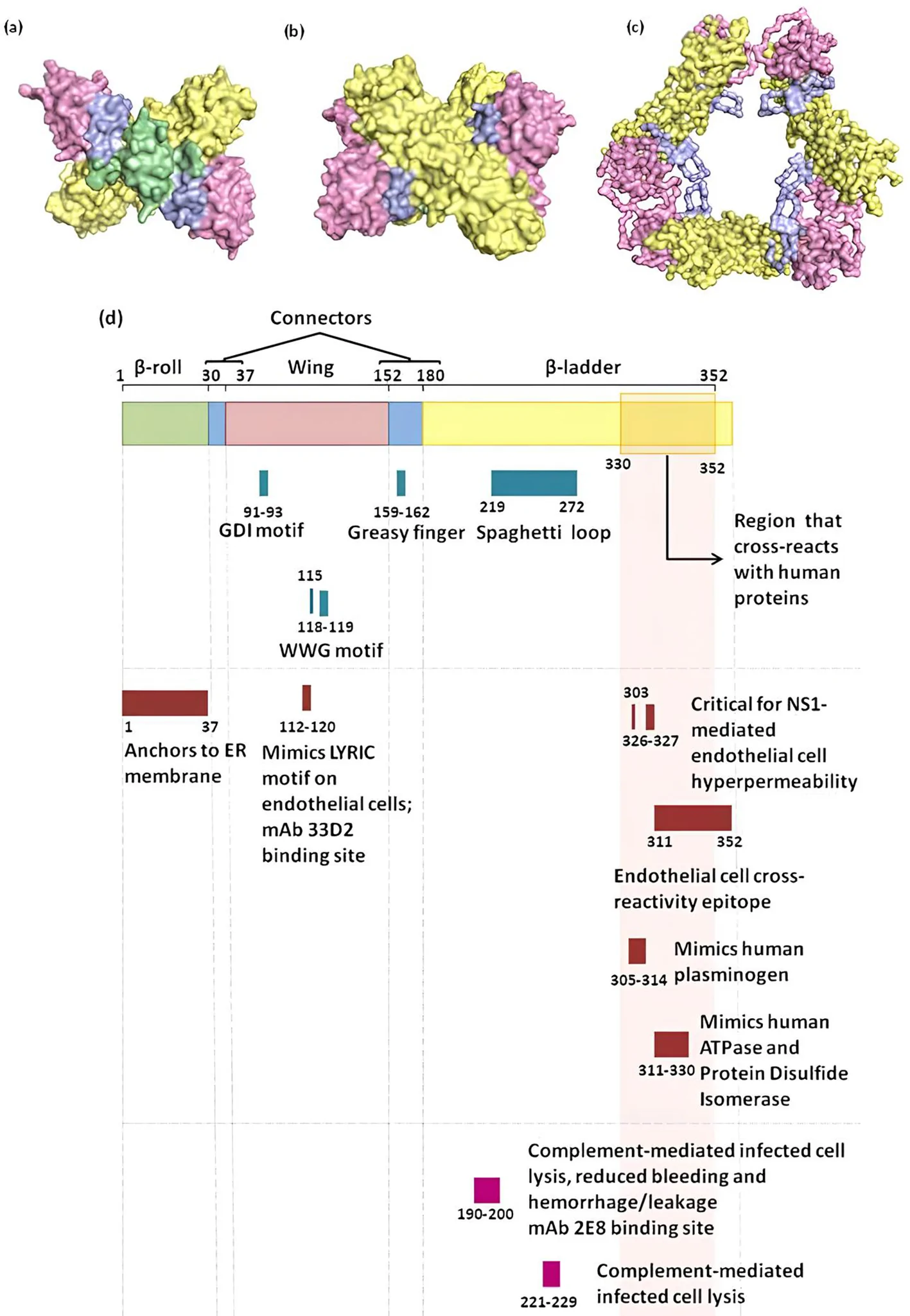
**Structural and functional domain architecture of dengue virus NS1 protein with mapped key residues.** (a) (b) & (c) This figure depicts the three-dimensional surface representation of the dengue virus non-structural protein 1 (NS1). (a) The dimeric form of NS1; PDB ID: 4O6B, (b) the tetrameric form; PDB ID: 8WBC and (c) the hexameric, secreted form; PDB ID: 7WUV are shown. Structural domains are color-coded to delineate functional architecture, with the β-roll domain (residues 1–29) displayed in pale green, connector regions (residues 30–37 and 152–180) in light blue, the wing domain (residues 38–151) in pink, and the elongated β-ladder domain (residues 181–352) in pale yellow. (d) This figure presents a schematic overview of the domain organization of the dengue virus non-structural protein 1 (NS1), highlighting its major structural and functional features. The β-roll domain (residues 1–29) is shown in green and plays a critical role in NS1 dimer formation. The wing domain (residues 38–151), displaced in pink, contributes to membrane interactions and immune modulation. Connector regions spanning residues 30–37 and 152–180 are illustrated in blue and provide structural flexibility between domains. The elongated β-ladder domain (residues 181–352), shown in yellow, constitutes the core scaffold of the NS1 dimer and hexamer assemblies. Structurally important regions are indicated by blue bars and include residues involved in dimerization, hexamerization, and membrane association, including the hydrophobic “greasy-finger” motif. Functionally important regions are highlighted using dark red and magenta bars. Dark red regions represent segments that mimic host proteins and are primarily associated with endoplasmic reticulum membrane binding and secretion. Magenta regions correspond to sites implicated in complement-mediated lysis of infected cells and immune recognition. Additionally, 330–352, highlighted by an orange box, marks the extreme C-terminal segment of NS1, adjacent to the host-protein-mimicry region (305–330) described above.

Given the widespread use of NS1 as a diagnostic biomarker, even modest, serotype-specific variation in NS1 may have implications for the sensitivity and consistency of NS1-based assays in hyperendemic settings. Understanding NS1 sequence diversity and selective pressures in the Indian context is therefore important for interpreting disease severity, immune recognition, and the continued reliability of NS1-based diagnostics.

## 2 Methodology

### 2.1 Data collection

All available dengue virus (DENV) whole-genome sequences were retrieved from the NCBI (National Centre for Biotechnology Information) and GISAID database. Only sequences with clearly defined collection dates were included in the study. Whole genome sequences from Indian isolates collected between 1956 and 2024, representing DENV-1 (*n* = 163), DENV-2 (*n* = 2546), DENV-3 (*n* = 171), and DENV-4 (*n* = 56) serotypes, were selected for further analysis.

### 2.2 Sequence alignment, haplotyping and recombination analysis

Whole-genome sequences were aligned using MAFFT Version 7, and the NS1 coding regions were subsequently extracted for downstream analyses (Katoh and Standley, 2013). To reduce redundancy in downstream analyses, identical NS1 nucleotide sequences were grouped into haplotypes prior to downstream analyses, ensuring a non-redundant representation of sequence diversity. Based on the variation in NS1 nucleotide sequences, the resulting haplotype counts were 67 for DENV-1, 953 for DENV-2, 64 for DENV-3, and 25 for DENV-4. Recombination analysis of NS1 haplotypes was performed using RDP5 software (Martin et al., 2021), employing a multi-method detection strategy to ensure high confidence in identifying recombinant events. Six independent algorithms, RDP, GENECONV, MaxChi, Chimaera, SiScan, and BootScan, were applied under default parameters. All sequences flagged as recombinants by two or more methods were removed from subsequent selection pressure and structural analyses to prevent artefacts arising from mosaic genome patterns.

### 2.3 Selection pressure analysis

NS1 sequences from all four DENV serotypes were independently analyzed for selection pressures using the MEME (Mixed Effects Model of Evolution), FEL (Fixed Effects Likelihood), SLAC (Single-Likelihood Ancestor Counting), and FUBAR (Fast Unconstrained Bayesian AppRoximation) models implemented in the HyPhy framework (Kosakovsky Pond et al., 2019). MEME detects episodic diversifying selection acting on a subset of lineages at individual sites, making it particularly suited for identifying transient adaptive events. In contrast, FEL and SLAC estimate site-specific selection by comparing non-synonymous and synonymous substitution rates averaged across the phylogeny, providing conservative evidence for pervasive selection but with reduced sensitivity under strong functional constraint. FUBAR applies a Bayesian framework to infer selection by integrating information across sites and branches, offering increased power to detect weak but consistent signals of selection while controlling for false positives. Convergence of signals across methods, where present, strengthens confidence in the identified sites, while sites detected by a single method are interpreted with appropriate caution, as discordance may reflect differences in the underlying assumptions and sensitivities of each approach. Sites meeting the predefined thresholds (*P* < 0.1 for MEME, FEL and SLAC; posterior probability ≥0.9 for FUBAR) are referred to throughout as showing evidence of diversifying selection. Non-synonymous amino acid substitutions were subsequently mapped onto the dimeric, tetrameric and hexameric NS1 protein structures obtained from RCSB-PDB (dimer: PDB ID 4O6B; tetramer: PDB ID 8WBC; hexamer: PDB ID 7WUV). Residue numbering refers to the aligned NS1 amino-acid positions and was maintained consistently across serotypes.

### 2.4 Predicted linear B-cell epitope mapping and structural analysis

Linear B-cell epitopes were predicted using the most represented haplotypes of NS1 sequence for each serotype (DENV-1- GenBank Accession No.- **MG721063.2**, DENV-2- GISAID Accession No.- EPI_ISL_18976830 (**hDenV2/India/DL-IGIB1130DF0834817D/2022**), DENV-3- GenBank Accession No.- **MN253132.2**, DENV-4- GenBank Accession No.- **PV138436.1**) using BepiPred-2.0 (Jespersen et al., 2017), and peptides with default scores ≥0.5 were selected. Conservation of these predicted epitopes was analyzed using the IEDB epitope conservancy tool (Bui et al., 2007). Diversifying amino acid substitutions were overlaid on NS1 structures to identify overlap between diversifying substitutions and predicted linear B-cell epitopes.

### 2.5 Structural mapping of diversifying sites on NS1-antibody complex and antibody interaction analysis

Diversifying sites falling within the resolved NS1 region of the PDB 7BSC were mapped onto the Fab-bound NS1 complex structure retrieved from the RCSB Protein Data Bank (PDB ID: 7BSC). This model represents a human broadly cross-reactive anti-NS1 antibody in complex with DENV-2 NS1 and therefore provides a biologically relevant, experimentally validated model for mapping antibody-interacting residues. Structural mapping was performed using PyMOL (The PyMOL Molecular Graphics System, Version 3.0 Schrödinger, LLC) to determine the minimum heavy-atom distance to antibody (Å) of each substitution mapped relative to the antibody-binding interface in the 7BSC model. To assess the potential impact of these substitutions on antigen-antibody interactions, each mutation represented in the resolved NS1 region of PDB: 7BSC was individually evaluated using the mCSM-AB web server tool (Pires and Ascher, 2016), which predicts changes in binding affinity (ΔΔG) resulting from amino acid substitutions. Predicted ΔΔG values were used to infer whether specific mutations were likely to enhance or reduce NS1-antibody binding. All tools and web-based resources are listed in Supplementary Table S3.

## 3 Results

### 3.1 Distinct NS1 haplotype distributions across DENV serotypes

A total of 2936 complete DENV genome sequences from India were retrieved from the NCBI GenBank and GISAID for downstream analyses, consisting of 163 DENV-1, 2546 DENV-2, 171 DENV-3, and 56 DENV-4 isolates. The uneven distribution of DENV sequences reflects differences in genomic surveillance and sequence availability across serotypes. In contrast, the persistently low number of DENV-4 sequences corresponded with its limited epidemiological presence, reflecting both reduced circulation and lower genomic surveillance efforts for this serotype.

The NS1 haplotype frequency plots in Fig. 2 exhibit haplotype richness with skewed frequency distribution across all four serotypes. Most NS1 haplotypes are represented by few sequences, alongside a limited number of dominant haplotypes present at higher frequencies. DENV-1 and DENV-3 represent a more even distribution of haplotypes however, DENV-2 shows one or a few highly dominant haplotypes with markedly higher frequencies than others with haplotype 87 comprising 351 sequences. DENV-4 consisted of the fewest haplotypes, consistent with its substantially smaller sample size; differences in surveillance intensity and circulation may also contribute. The persistence of dominant NS1 haplotypes across datasets suggests consistent functional constraints although sampling structure may also contribute to the observed frequency distribution.

**Fig. 2:**
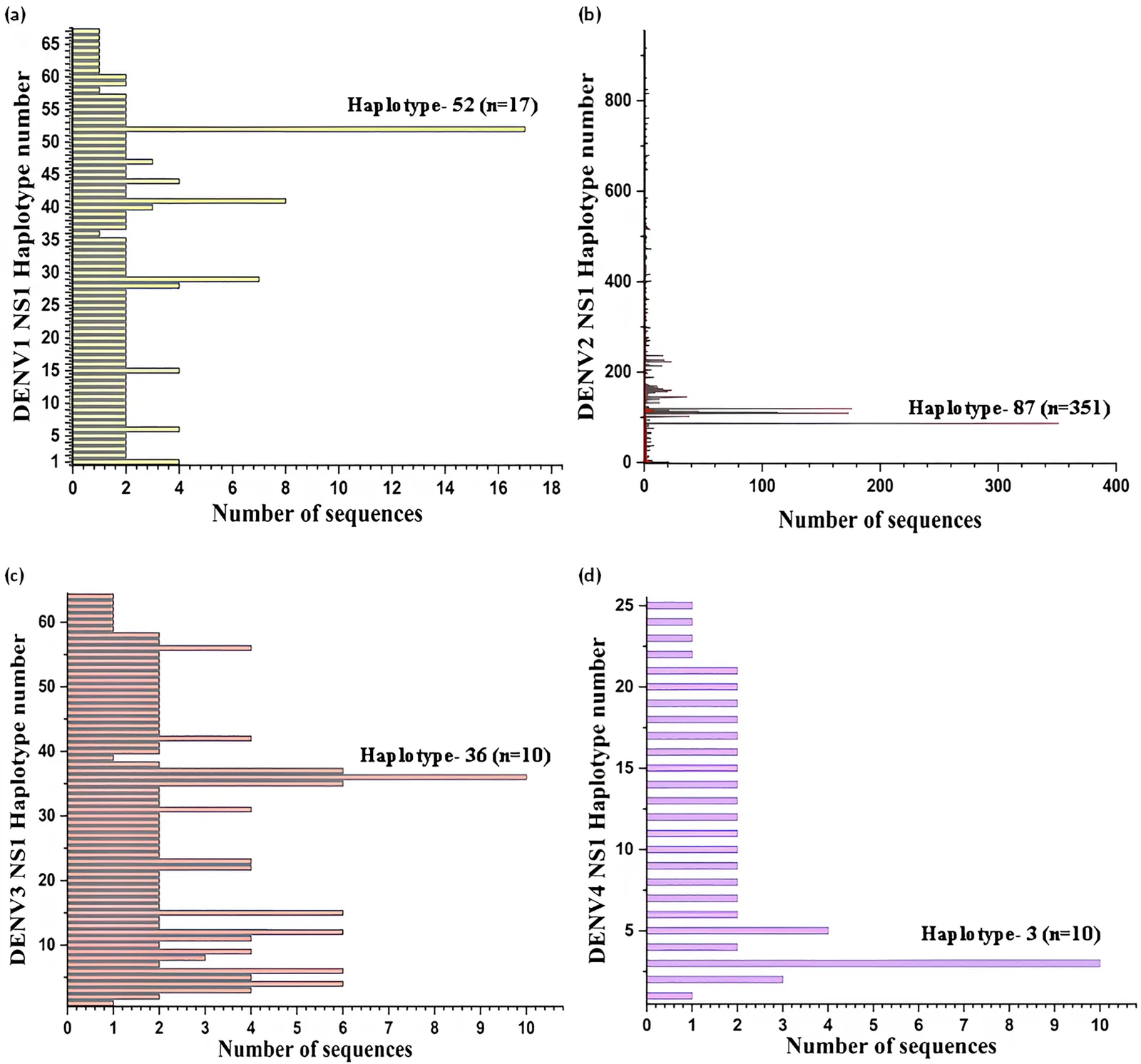
**Haplotype frequency distribution of dengue virus (DENV) NS1 sequences across all four serotypes. (a) DENV-1; (b) DENV-2; (c) DENV-3; (d) DENV-4.** This figure illustrates the distribution and frequency of NS1 haplotypes identified across the four dengue virus serotypes: (a) DENV-1 (green), (b) DENV-2 (red), (c) DENV-3 (pink), and (d) DENV-4 (purple). Each horizontal bar represents a unique NS1 haplotype, with the x-axis indicating the number of sequences supporting that haplotype and the y-axis denoting the corresponding haplotype number. Across all serotypes, a large proportion of haplotypes are represented by a small number of sequences, reflecting the presence of numerous low-frequency or rare variants. In contrast, a limited number of haplotypes show disproportionately high frequencies, indicating dominant or widely circulating NS1 variants within each serotype. DENV-2 yielded the largest number of distinct NS1 haplotypes and candidate diversifying sites; however, these comparisons are strongly influenced by the substantially larger DENV-2 sample size. DENV-1 and DENV-3 also display moderate haplotype diversity with a mix of rare and moderately frequent haplotypes, whereas DENV-4 shows comparatively fewer haplotypes and lower overall frequencies, indicative of reduced diversity. Collectively, these patterns highlight serotype-specific differences in NS1 genetic variability and evolutionary dynamics, suggesting that distinct selective pressures, transmission histories, and population bottlenecks shape the haplotype structure of NS1 across dengue virus serotypes.

### 3.2 Selection analyses suggest strong constraint with limited diversification in NS1

To identify sites under diversifying selection, the NS1 gene sequences of all four dengue virus serotypes were analyzed using MEME, FEL, SLAC (*p*-value <0.1) and FUBAR models Prob[α < β] (≥0.9). Across MEME, FEL, and SLAC, especially under sparse sampling, low sequence divergence and strong purifying selection acting on NS1, a relaxed significance threshold of *p* < 0.1 was used to improve sensitivity and reduce false negatives while interpreting results in conjunction with multiple complementary methods. 14 sites showing evidence of diversifying selection, involving the following amino-acid substitutions, S94N/Y/A, D278N, D288E/N, K324R in DENV-1, S80T, I96V, T109I/A, S128L/F/P/H, N222S, N234M/D, Y260I, I264T in DENV-2, Y175N, I287V in DENV-3, and none in DENV-4, were detected (Supplementary Table S4). Of these 14 diversifying sites, only four - S94N and K324R in DENV-1, and S80T and I96V in DENV-2, were supported by two or more of the four selection-detection methods, while the remaining ten sites (D278N and D288E/N in DENV-1; T109I/A, S128L/F/P/H, N222S, N234M/D, Y260I and I264T in DENV-2; and Y175N and I287V in DENV-3) were flagged by a single method, predominantly MEME, at the relaxed p<0.1 threshold. Most of these sites fall in the β-ladder and wing domains, which are known to be surface exposed functional domains. The percent frequency of mutated amino acids is also shown in Supplementary Table S4. Positively selected codons generally exhibited low to moderate frequency, with several sites showing less than 10% frequency across analyzed sequences. Notably, certain diversifying sites retained high frequency in the dataset. For instance, the K324R substitution was present in 77% of DENV-1 sequences, and S80T in 89.3% of DENV-2 sequences. Such high frequencies suggest that these substitutions may represent near-fixed or lineage-defining variants rather than sites under continued episodic diversification, a possibility explored further in the Discussion. The number and distribution of diversifying NS1 residues varied among serotypes, with DENV-2 exhibiting a greater number of diversifying sites compared to DENV-1 and DENV-3.

These sites were also mapped on the dimeric, tetrameric and hexameric structures of NS1 depicting several residues showing evidence of diversifying selection, localizing to exposed regions of NS1 oligomers, potentially influencing antibody accessibility and host protein interactions as shown in Fig. 3. Diversifying sites are largely excluded from NS1 oligomerization interfaces, including regions critical for dimer stabilization and hexamer formation.

**Fig. 3:**
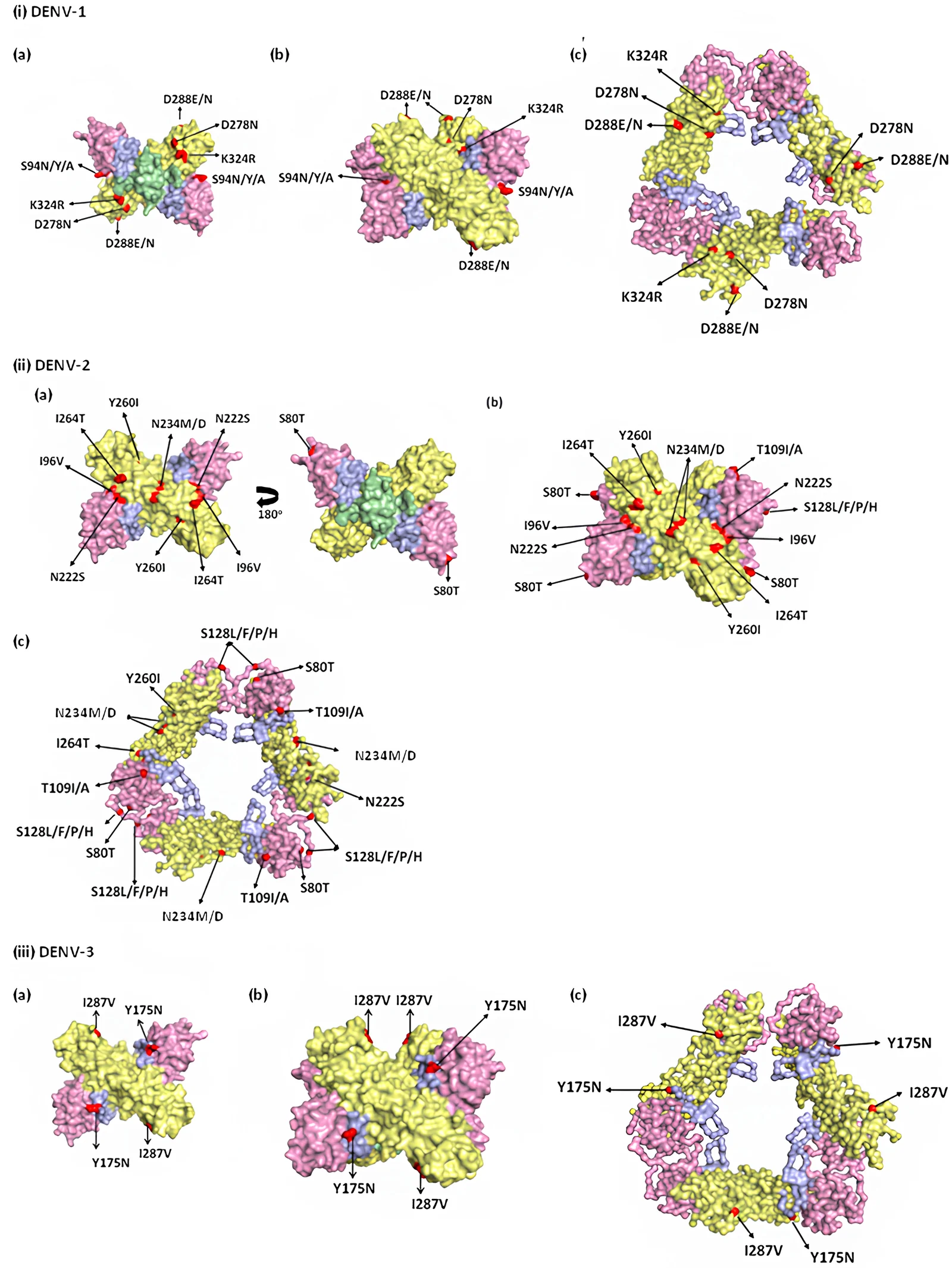
**Structural mapping of NS1 domains and sites showing evidence of diversifying selection on the oligomeric assemblies of dengue virus NS1 (i) DENV-1; (ii) DENV-2; (iii) DENV-3.** This figure depicts the three-dimensional surface representation of the dengue virus non-structural protein 1 (NS1): (i) DENV-1, (ii) DENV-2, (iii) DENV-3, highlighting the approximate spatial distribution of structural domains within oligomeric NS1 assemblies. The (a) dimeric form of NS1; PDB ID: 4O6B, (b) tetrameric form; PDB ID: 8WBC and (c) hexameric, secreted form; PDB ID: 7WUV are shown to illustrate conformational differences associated with different functional states of NS1. Structural domains are color-coded to delineate functional architecture, with the β-roll domain (residues 1–29) displayed in pale green, connector regions (residues 30–37 and 152–180) in light blue, the wing domain (residues 38–151) in pink, and the elongated β-ladder domain (residues 181–352) in pale yellow. Residues identified as showing evidence of diversifying selection through codon-based evolutionary analyses (MEME, FEL, SLAC, and FUBAR) are mapped onto the NS1 surface in red. These diversifying sites are distributed across multiple domains rather than being confined to a single region, with several residues exposed on the protein surface. Their surface exposure provides a structural context for considering potential effects on antibody accessibility or host interactions. Overall, this structural mapping links evolutionary signatures of diversifying selection with functionally relevant and solvent-exposed regions of NS1 across DENV serotypes.

### 3.3 Overlap of sites showing evidence of diversifying selection with structurally accessible predicted linear B-cell epitopes

Linear B-cell epitopes (BCEs) were identified/predicted using BepiPred2.0 with the highest number of BCEs predicted in the NS1 sequence of DENV-1 (12 epitopes) (Supplementary Table S5). Mapping of predicted linear B-cell epitopes overlapping with residues showing evidence of diversifying selection, shown in Fig. 4 revealed that evidence of diversifying selection was not uniformly distributed across all predicted epitopes but was restricted to specific BCEs. In DENV-1, S94N/Y/A overlap with epitope B1 which is 78.69% conserved among the dataset. However, D278N and D288E/N overlap epitope B10 and K324R overlaps B11, each being 1.64% and 22.95% conserved. This suggests coexistence of both conserved and highly variable antibody-targeted regions within the protein. In contrast, in DENV-2 and DENV-3, the overlap of diversifying sites predominantly with highly conserved predicted linear B-cell epitopes (80–98% conservation) suggests serotype-specific pattern of immune constraint on the regions predicted to have linear B-cell epitope potential (Supplementary Table S6). The DENV-2 N222S substitution did not overlap with any predicted linear B-cell epitope, as it was located at residue 222, between epitopes B5 (residues 217–221) and B6 (residues 223–244) (Supplementary Tables S5 and S6). Notably, several predicted BCEs displayed high levels of conservation among the Indian dataset (∼80–98%; Supplementary Table S5), consistent with episodic selection acting on rare evolutionary branches rather than population-wide fixation.

**Fig. 4:**
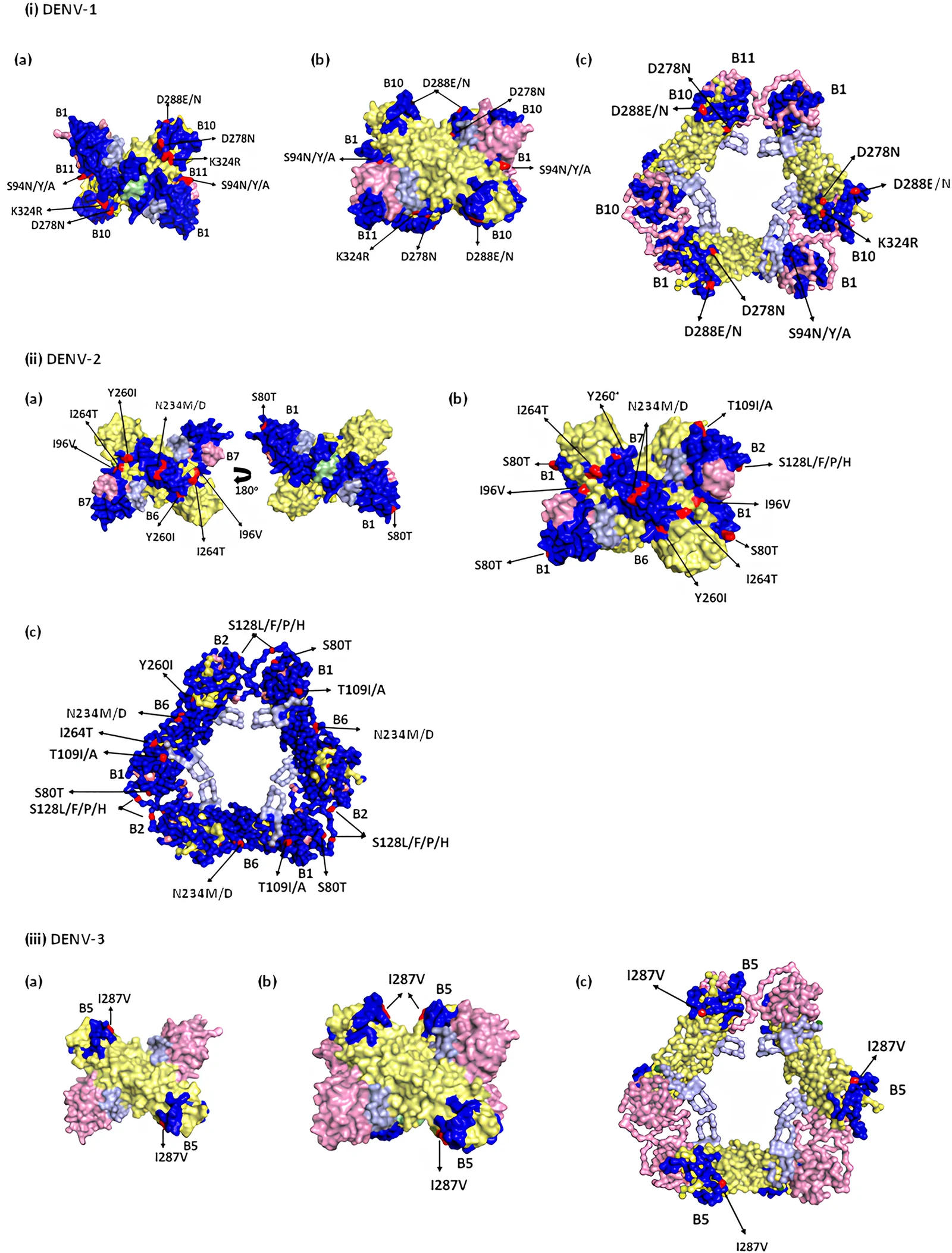
**Structural mapping of sites showing evidence of diversifying selection NS1 residues overlapping predicted linear B-cell epitopes in dimeric, tetrameric and hexameric assemblies of DENV NS1.** This figure depicts the three-dimensional surface representation of the dengue virus non-structural protein 1 (NS1): (i) DENV-1, (ii) DENV-2, (iii) DENV-3, highlighting the approximate spatial distribution of structural domains within oligomeric NS1 assemblies. The (a) dimeric form of NS1; PDB ID: 4O6B, (b) tetrameric form; PDB ID: 8WBC and (c) hexameric, secreted form; PDB ID: 7WUV are shown to illustrate conformational differences associated with different functional states of NS1. Structural domains are color-coded to delineate functional architecture, with the β-roll domain (residues 1–29) displayed in pale green, connector regions (residues 30–37 and 152–180) in light blue, the wing domain (residues 38–151) in pink, and the elongated β-ladder domain (residues 181–352) in pale yellow.  Linear B-cell epitopes predicted using BepiPred-2.0 are highlighted in blue, indicating regions with immunogenic potential. Diversifying residues identified through codon-based evolutionary analyses are overlaid in red when they coincide with these predicted epitopes. Together, this mapping highlights a convergence of diversifying selection and regions predicted to have linear B-cell epitope potential, consistent with immune-associated selection shaping NS1 sequence variation.

### 3.4 Diversifying NS1 sites are spatially distal from the primary antibody–binding interface

To assess the potential impact of amino acid variability on antibody recognition, all sites under diversifying selection were mapped onto the NS1-antibody complex structure (PDB ID: 7BSC) as shown in Fig. 5. Using the DENV-2 NS1–1G5.3 complex as a structural reference, none of the mapped diversifying sites within the resolved region directly overlapped the antibody-contacting interface. Structural mapping of amino acid substitutions onto the NS1 antigen-antibody complex demonstrated that all variant residues were located outside the direct antibody-contacting interface, with the closest residues positioned proximal to, but not overlapping, the interface. Minimum heavy-atom distance measurements, calculated using PyMOL, ranged from 11 to 42 Å. Among DENV-1 variants, the K324R substitution was closest to the antibody surface (11 Å), followed by D278N (15 Å). In DENV-2, substitutions including N222S, N234M/D, Y260I, and I264T were positioned at comparatively greater distances from the binding interface, measuring 26, 42, 29, and 23 Å, respectively. The DENV-3 substitution I287V was located 15 Å away from the antibody-binding surface in the NS1 structure. To quantitatively evaluate their impact, individual mutations were analyzed using mCSM-AB to predict changes in antigen-antibody binding affinity (ΔΔG) (Supplementary Table S7). In DENV-1, the K324R substitution exhibited increase in binding affinity (ΔΔG = +0.231 kcal/mol) and was predicted to reduce binding affinity (ΔΔG = −0.78 kcal/mol) however, these predicted changes are relatively small and should not be interpreted as evidence of a substantial change in antibody binding without experimental validation. In contrast, DENV-2 N222S mutation was predicted to modestly reduce antibody binding affinity, with ΔΔG value of −0.279 kcal/mol. Similarly, in DENV-3, I287V mutation decreased binding affinity (ΔΔG = −0.381 kcal/mol). Collectively, these findings suggest that although diversifying sites do not directly participate in antibody contact, several substitutions proximal to the binding interface may exert subtle, context-dependent effects on antigen-antibody interactions.

**Fig. 5:**
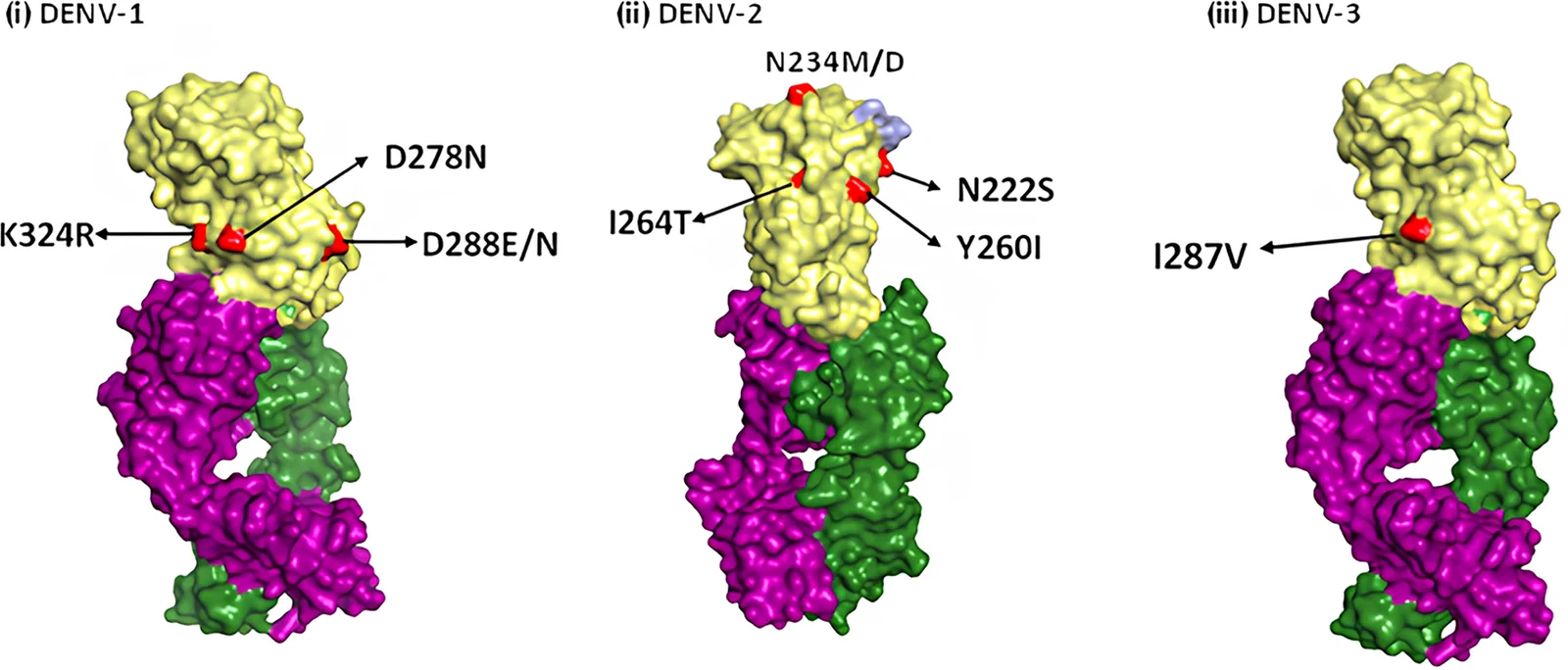
**Structural localization of NS1 sites showing evidence of diversifying selection relative to the NS1-antibody interface.** This figure presents surface representations of the dengue virus NS1–antibody (human 1G5.3 Fab) complex (PDB ID: 7BSC) to illustrate the approximate spatial positioning of diversifying NS1 residues relative to the antibody-binding interface for (i) DENV-1, (ii) DENV-2, and (iii) DENV-3 using the DENV-2 NS1–1G5.3 Fab complex (PDB ID: 7BSC) as the structural reference. The NS1 protein is shown with the connector region highlighted in light blue and the extended β-ladder domain in pale yellow, providing structural context for regions implicated in immune recognition. The bound antibody is represented with the heavy chain colored purple and the light chain colored green. Residues identified as being under diversifying selection through codon-based evolutionary analyses are mapped onto the NS1 surface in red and labeled to emphasize their proximity to the antibody contact surface. Notably, the 7BSC structure encompasses the second connector region of NS1 (residues 171–180) along with the C-terminal portion of the protein spanning the β-ladder domain, thereby capturing key immunologically relevant segments of NS1. Sites S94N/Y/A in DENV-1, S80T, I96V, T109I/A, S128L/F/P/H in DENV-2 and Y175N in DENV-3 were not mapped on the NS1 region because they lie outside the 7BSC coverage. Positively selected residues are located outside the direct antibody-contacting interface.

## 4 Discussion

Most global investigations of dengue virus evolution have focused on the envelope (E) protein, where immune-driven adaptation has been extensively characterized (Katzelnick et al., 2021). In contrast, NS1 has received comparatively less attention, particularly in geographically resolved datasets, limiting insights into how immune pressure shapes non-structural antigens at regional scales. Globally aggregated dengue virus datasets, while valuable for broad evolutionary inference, may obscure region-specific selective pressures operating in hyperendemic settings where multiple serotypes co-circulate and population immunity is repeatedly shaped by sequential infections. By leveraging an India-specific, serotype-resolved NS1 dataset, this study captures evolutionary patterns emerging under sustained hyperendemic transmission. Our approach with serotype-wise haplotype-based curation of NS1 sequences of Indian isolates depicts relatively greater haplotype richness (*n* = 953) in DENV-2 as compared to other serotypes, in which the haplotype distribution was moderate. This coincides with larger number of sequences for DENV-2 (*n* = 2546). Moreover, the presence of only a few dominant haplotypes within each serotype may reflect functional constraints of NS1 protein. DENV-4 showed the lowest observed haplotype richness in this dataset, although this may partly reflect its substantially smaller sample size. Further, to assess selection pressures acting on NS1, we employed codon-based methods, MEME, FEL, SLAC, and FUBAR, each capturing distinct evolutionary signals. The relatively small number of sites showing evidence of diversifying selection is compatible with substantial evolutionary constraint across NS1, although sampling and phylogenetic power also influence these estimates. DENV-2 had the most candidate sites, but this is confounded by its much larger sample size. The much larger number of available DENV-2 sequences may reflect differences in circulation, genomic surveillance and/or sampling intensity. The S80T mutation is present in 89.3% of sequences within the dataset while other mutations showed a frequency of 0.1–8%. Mutations with high frequency may reflect fixed or nearly-fixed substitutions, rather than indicating ongoing episodic diversification. In contrast, the reduced number of diversifying sites in DENV-1 and DENV-3 is compatible with stronger evolutionary constraint, although differences in sampling and statistical power may also contribute. However, high frequency of K324R mutation in DENV-1 (77%) may be compatible with persistence of the variant in the sampled population. A study by Ogire et al. (2021) revealed that K324R, together with K272R, has been experimentally implicated in enhanced NS1 stability and secretion in Huh7 cells (Ogire et al., 2021). Additionally, conservation of residues of important glycosylation sites like N130 and N207 suggests their significance in the functionality of the protein and thus presence of evolutionary constraint (Tajima et al., 2008). Structural mapping revealed that most diversifying residues localize to surface-exposed regions of the wing and β-ladder domains, which contain several reported antibody-recognized regions. These patterns can suggest that NS1 evolution balances the need to preserve essential oligomeric and functional interactions while allowing some plasticity in immune-exposed regions. Conversely, no sites under episodic diversification were found in NS1 sequences of DENV-4 possibly due to sampling bias in addition to true epidemiology of DENV-4 in India. Although prediction of linear BCEs do not account for conformational epitopes, consistent with the experimental literature, most predicted linear B-cell epitopes fall in the wing and β-ladder domains of NS1 which are also the most exposed regions of the protein. Some BCEs also overlapped with an immunodominant region near the C-terminus of the protein. Candidate sites were localized to a subset of predicted BCEs. Evidence of diversifying selection was confined to a limited subset of B-cell epitopes, indicating localized rather than widespread diversification. DENV-2 showed the highest overlap with conserved epitopes (80–98%), suggesting stronger functional constraint, whereas DENV-3 exhibited diversification restricted to a single epitope. Crucially, several predicted BCEs lacked overlap with diversifying residues despite being surface-exposed, indicating the existence of immunogenic yet evolutionarily conserved regions. Such regions may represent conserved antibody-targeted regions that warrant further investigation for their potential functional and immunological relevance.

For the diversifying substitutions represented in the resolved region of PDB 7BSC, the minimum heavy-atom distances from the antibody surface ranged from 11 to 42 Å suggesting that these sites are unlikely to contribute to direct antibody binding. Instead, absence of direct overlap of the diversifying sites with antibody-interacting surface raises the possibility that they may exert indirect effects through long-range structural or conformational mechanisms, potentially influencing epitope accessibility. mCSM-AB predictions do not provide evidence for a substantial alteration in 1G5.3 binding, although experimental measurements would be required to determine whether any substitution affects antibody recognition.

Based on the observed serotype-specific selection patterns and epitope localization within NS1, this study generates several testable predictions. NS1-based diagnostic assays relying on conserved β-ladder epitopes may have potential to show improved cross-serotype sensitivity, whereas assays targeting regions under diversifying selection, particularly in DENV-2, may exhibit serotype-biased performance. Although the predicted substitutions did not show direct overlap with antibody-binding interfaces and showed minimal effects on binding free energy, they may indirectly influence antigenicity through conformational or dynamic changes having potential effects on ELISA sensitivity and related antibody binding assays. Importantly, these *in silico* predictions require experimental validation using recombinant NS1 variants, binding kinetics assays, and clinical serum panels to determine their functional impact on antibody recognition and diagnostic performance.

The study also has some limitations that should be considered while interpreting the findings. The analysis relies on publicly available NS1 sequences, and although haplotype-based curation was employed to reduce redundancy, uneven geographic and temporal sampling, particularly across Indian regions and serotypes, may influence estimates of diversity and site-specific selection. Selection pressure inferences were based on codon-based phylogenetic models that are sensitive to sequence number, alignment quality, and phylogenetic signal. Thus, variation in the number and availability of Indian sequences across the four DENV serotypes in public databases should be considered while interpreting the serotype-specific haplotype and selection analyses, particularly because unequal sample sizes (especially in DENV-4) may influence estimates of genetic diversity and the power to detect sites showing evidence of diversifying selection. While multiple complementary methods were applied to improve robustness, subtle signals of selection may remain undetected or reflect episodic events on limited branches. Additionally, B-cell epitope mapping was restricted to predicted linear epitopes using BepiPred-2.0 and therefore does not capture conformational or discontinuous epitopes, which are highly relevant for antibody recognition of NS1 oligomers. Structural and binding affinity analyses were computational and predictive in nature; the inferred effects of amino-acid substitutions on antibody interaction require experimental validation using biophysical or immunological assays.

Despite these limitations, our study raises important considerations regarding the performance of NS1-based assays in hyperendemic settings such as India. Although NS1 is generally conserved and widely used as a diagnostic biomarker, localized sequence diversification, particularly within or proximal to immuno-epitopic regions, may influence antigen recognition by assay antibodies in a serotype-dependent manner. From a vaccine perspective, the identification of highly conserved, antibody-accessible NS1 regions across multiple serotypes may warrant further investigations as candidate immunogenic targets, particularly in vaccine designs that aim to complement envelope-based immunity while minimizing the risk of ADE. Conversely, regions exhibiting greater variability, especially those overlapping predicted BCEs, may be less attractive as candidates for broadly conserved vaccine targets, pending experimental validation. Consequently, these findings highlight the importance of continued surveillance of NS1 sequence diversity to ensure the robustness and broad applicability of NS1-based diagnostic platforms and vaccine design across all circulating dengue serotypes.

## CRediT authorship contribution statement

**Khushi Khera:** Writing – original draft, Methodology, Formal analysis, Data curation, Conceptualization. **Sanjeev Kumar:** Writing – review & editing, Methodology, Conceptualization. **Anmol Chandele:** Writing – review & editing, Conceptualization. **Amit Sharma:** Writing – review & editing, Supervision, Methodology, Conceptualization.

## Ethical approval statement

No ethical approval was required for this study.

## Funding source

This work was funded by a 10.13039/100010269Wellcome Trust grant (328498/Z/25/Z).

## Declaration of competing interest

The authors declare that they have no known competing financial interests or personal relationships that could have appeared to influence the work reported in this paper.

## Data Availability

Data will be made available on request.
