# Supplementary material for: Immune-driven serotype-specific evolution of dengue virus NS1: Insights from structural and epitope analyses of Indian isolates
Source: Infect Genet Evol. 2026 Nov;145:106021. doi: 10.1016/j.meegid.2026.106021 (PMC13748514; doi:10.1016/j.meegid.2026.106021)
Supplement: Supplementary file 1 [file mmc1.docx]

**Supplementary Data**

**Supplementary Table S1 –** Functional annotation of Dengue virus NS1 domains and key residues.

| **Amino acid position** | **Domain** | **Role** | **Reference** |
| --- | --- | --- | --- |
| 1-29 | β-roll | Two β-rolls interlock together to form a dimer | [1] |
| 30-37, 152-180 | Connectors | Connects wing domain to the central β-roll and β-ladder domains | [1] |
| 38-151 | Wing | Has glycosylation site; Attached to central β-ladder; Have two subdomains: an α/β subdomain and a connector subdomain, which packs against the β-roll and links the wing to the central β-ladder domain through an internal disulfide bond. | [1] |
| 181-352 | β-ladder | Has glycosylation site; Facilitates head to head dimerization of two NS1 monomers; together with β-roll, makes the dimerization interface. | [1] |
| 159-162 | Greasy finger | Interacts with viral proteins NS4A/NS4B | [2] |
| 219-272 | Spaghetti loop | Forms a long loop that lacks secondary structure, ordered by 57 hydrogen bonds | [3] |
| 1-37 |  | Anchored to the Endoplasmic Reticulum membrane through the hydrophobic N-terminal β-roll–Connector | [3] |
| 91-93 | GDI motif | Engages with endothelial cells and platelets which triggers platelet activation and endothelial hyperpermeability | [2] |
| 112-120 |  | Critical for endothelial cell binding and platelet binding/activation; Mimics to LYRIC motif on endothelial cells, mAb 33D2 binding site, Contains WWG motif (115, 118-119) | [3], [4] |
| 190-200, 221-229 |  | Complement mediated lysis of infected cells, mAb 2E8 binding site | [4], [5] |
| 305-314 |  | Mimics to Human plasminogen | [6] |
| 303, 326-327 |  | Critical for NS1 mediated endothelial hyperpermeability | [3] |
| 311-330 |  | Mimics to Human ATPase, HSp-60 and Protein Disulfide Isomerase | [6] |
| 311-352 |  | Endothelial cell cross-reactivity epitope | [3] |

**Supplementary Fig. S1 -** Comprehensive epitope and immunodominant landscape of Dengue virus NS1 protein mapped onto its domain architecture.

(a) This figure presents a comprehensive linear schematic of the Dengue virus NS1 protein, integrating structural domain organization, predicted B-cell epitopes, experimentally reported epitopes, conservation patterns, cross-reactivity, and sites under positive selection. The NS1 domain architecture is shown at the top, with the β-roll domain depicted in green, connector regions in blue, the wing domain in pink, and the extended β-ladder domain in yellow. (i) Known experimentally validated epitopes are highlighted in purple, with their epitope names and residue positions indicated. (ii)-(iv) highlights different epitopes either experimentally validated or computationally extracted corresponding to different monoclonal antibodies against NS1. (v) and (vi) highlights regions showing high antibody response. (vii) Conserved epitopes shared across multiple sequences are shown in blue, reflecting regions of high sequence conservation and potential diagnostic relevance. (viii) Epitopes reported to be cross-reactive with other dengue serotypes and possible mAb 4H2 target regions are marked in orange, along with their amino acid coordinates, emphasizing regions that may contribute to serotype cross-recognition. (b) (i)-(viii) The panel of figures presents the same epitope regions marked in (a) with representative colors mapped onto the dimeric model of NS1 (PDB ID: 4O6B).

**Supplementary Table S2 –** Characterization of epitope sites identified in Dengue virus NS1 protein

| **Epitope sites** | **Sequence** | **Significance** | **Reference** |
| --- | --- | --- | --- |
| 111-121 (LX1) | 111-LRYSWKTWG-119; 113-YSWKTWGKA-121 | mAb 4H3.4 and mAb 3A5.4 target region | [7] |
| 25-33 (LD2) | 25-VHTWTEQYK-33 | mAb 1H7.4 and mAb 5H4.4 target region | [7] |
| 61-69 (24A) | 61-TRLENLMWK-69 | mAb 5H4.3 target region | [7] |
| 299-309 (24C) | 299-RTTTASGKL-307/301-TTASGKLIT-309 | mAb 5H5.4 and 1G5.3 target region | [7] |
| 27-33, 97-116, 125-134, 143-151, 170-178, 289-297 | 27-TWTEQYKFQ-35, 97-MQAGKRSLRPQPTELKYSWK-116, 125-STESHNQTFL-134, 143-CPNTNRAWN-151, 170-KLREKQDVF-178, 289-EDCGNRGPS-297 | mAb 1H7.4 target region | [8] |
| 111-128, 233-241, 282-291, 303-311 | 111-LKYSWKTWGKAKMLSTES-128, 233-SNGVLESEM-241, 282-GTTVVVTEDC-291, 303-ASGKLITEWC-311 | mAb 1G5 target region | [8] |
| 27-36, 110-118, 124-132, 249-264 | 27-TWTEQYKFQP-36, 110-ELKYSWKTW-118, 124-LSTESHNQT-132, 249-GPVSQHNYRPGYYTQT-264 | mAb GUS2 target region | [8] |
| 1-24, 57-77, 233-245 | 1- MNSRSTSLSVSQVLVGIVTLYLGV-25, 57- TEQYKFQPESPSKLASAIQKA-77, 233- ALNDTWKIEKASF-245 | High IgG and IgM responses in human ELISA | [9] |
| 36-45, 187-196 | 36-PESPSKLASA-45; 187-AIKDNARAVHA-196 | Possible dominant B-cell epitopes | [10] |
| 56-71, 114-119, 154-170, 193-204, | 56-GIRSTTRLENVMWKQI-71, 154-EVEDYGPGVFTTNIWLK-170, 193-AVHADMGYWIES-204, 325-GEDGCWYGMEIRP-337 | Highly conserved among other serotypes | [11] |
| 127-143 | 127- ELHNQTFLIDGPETAEC-143 | Cross-reactive to other serotypes | [12] |

**Supplementary table S3 –** Tools/web-based resources used and their objectives

| **S. No.** | **Tool/website Used** | **Objective** | **Reference** |
| --- | --- | --- | --- |
| 1. | National Centre for Vector Borne Diseases Control (NCVBDC) | Source of official epidemiological data on dengue incidence and distribution in India. | [13] |
| 2. | MAFFT Version 7 | Used for multiple sequence alignment of DENV NS1 nucleotide and amino acid sequences. | [14] |
| 3. | RDP5 software | Employed to detect and statistically validate potential recombination events in NS1 sequences. | [15] |
| 4. | HyPhy framework | Applied to estimate site-specific selection pressures using codon-based evolutionary models. | [16] |
| 5. | BepiPred 2.0 | Utilized to predict linear B-cell epitopes within the NS1 protein sequence. | [17] |
| 6. | IEDB epitope conservancy tool | Used to evaluate the degree of conservation of predicted B-cell epitopes across DENV isolates. | [18] |
| 7. | mCSM-AB web server tool | Applied to assess the impact of amino acid substitutions on NS1–antibody binding affinity. | [19] |
| 8. | PyMOL Molecular Graphics System | Used for three-dimensional structural visualization and mapping of mutations and epitopes on NS1 models. | [20] |

**Supplementary Table S4 –** Positively selected sites in NS1 sequences of all Dengue virus (DENV) serotypes from the Indian dataset.

| **Serotype** | **Codon position** | **Reference amino acid** | **Mutated amino acid** | **P-values (<0.1)** | | | **FUBAR prob[α < β] (>0.9)** | **Structural domain of NS1** | **Mutant amino acid frequency (%)** |
| --- | --- | --- | --- | --- | --- | --- | --- | --- | --- |
|  |  |  |  | **MEME** | **FEL** | **SLAC** |  |  |  |
| DENV-1 | 94 | S | N | 0.097 | - | - | 0.925 | Wing | 7% |
|  |  |  | Y |  |  |  |  |  | 2% |
|  |  |  | A |  |  |  |  |  | 2% |
|  | 278 | D | N | 0.077 | - | - | - | β-ladder | 30% |
|  | 288 | D | E | 0.087 | - | - | - | β-ladder | 2% |
|  |  |  | N |  |  |  |  |  | 2% |
|  | 324 | K | R | 0.078 | 0.0579 | - | 0.985 | β-ladder | 77% |
| DENV-2 | 80 | S | T | - | 0.087 | 0.0978 | - | Wing | 89.3% |
|  | 96 | I | V | 0.0498 | - | 0.0347 | 0.9067 | Wing | 1.2% |
|  | 109 | T | I | 0.0255 | - | - | - | Wing | 0.1% |
|  |  |  | A |  |  |  |  |  | 0.5% |
|  | 128 | S | L | 0.0005 | - | - | - | Wing | 8.5% |
|  |  |  | F |  |  |  |  |  | 1% |
|  |  |  | P |  |  |  |  |  | 7.3% |
|  |  |  | H |  |  |  |  |  | 0.8% |
|  | 222 | N | S | - | - | - | 0.9070 | β-ladder | 7.1% |
|  | 234 | N | M | 0.0093 | - | - | - | β-ladder | 0.1% |
|  |  |  | D |  |  |  |  | β-ladder | 0.2% |
|  | 260 | Y | I | 0.0332 | - | - | - | β-ladder | 0.1% |
|  | 264 | I | T | 0.0364 | - | - | - | β-ladder | 9.3% |
| DENV-3 | 175 | Y | N | 0.098 | - | - | - | Connector | 2% |
|  | 287 | I | V | 0.092 | - | - | - | β-ladder | 3% |

**Supplementary Table S5 -** Serotype-wise predicted B-cell epitope sequences predicted with BepiPred2.0 with start and end coordinates in NS1 protein, domains of NS1 covered by the epitope and percent conservation of predicted epitopes in the Indian dataset.

| **Serotype** | **B-cell epitope number** | **Epitope coordinates** | | **Epitope sequence** | **Domains of NS1** | **Conservation of epitopes in Indian dataset (%)** |
| --- | --- | --- | --- | --- | --- | --- |
|  |  | **Start** | **End** |  |  |  |
| **DENV-1** | **B1** | **6** | **96** | **INWKGRELKCGSGIFVTNEVHTWTEQYKFQADSPKRLSAAIGKAWEEGVCGIRSATRLENIMWKQISNELNHILLENDMKFTVVVGDANGI** | **β-roll/wing** | **78.69%** |
|  | B2 | 109 | 121 | MEHKYSWKSWGKA | Wing | 96.72% |
|  | B3 | 123 | 136 | IIGADTQNTTFIID | Wing | 54.10% |
|  | B4 | 142 | 154 | ECPDDQRAWNIWE | Wing/connector | 91.80% |
|  | B5 | 172 | 214 | RDSYTQMCDHRLMSAAIKDSKAVHADMGYWIESEKNETWKLAR | Connector/ β-ladder | 73.77% |
|  | B6 | 218 | 221 | IEVK | β-ladder | 98.365% |
|  | B7 | 224 | 242 | IWPRSHTLWSNGVLESEMI | β-ladder | 81.975% |
|  | B8 | 254 | 256 | HNY | β-ladder | 98.36% |
|  | B9 | 265 | 272 | AGPWHLGK | β-ladder | 100.00% |
|  | **B10** | **276** | **315** | **DFDLCEGTTVVVDEHCGNRGASLRTTTVTGKIIHEWCCRS** | **β-ladder** | **1.64%** |
|  | **B11** | **322** | **328** | **RFKGEDG** | **β-ladder** | **22.95%** |
|  | B12 | 342 | 348 | EENLVRS | β-ladder | 91.80% |
| **DENV-2** | **B1** | **6** | **116** | **VSWKNKELKCGSGIFITDNVHTWTEQYKFQPESPSKLASAIQKAHEEGICGIRSVTRLENLMWKQITPELNHILTENEVKLTIMTGDIKGIMQAGKRSLRPQPTELKYSWK** | **β-roll/Wing** | **82.79%** |
|  | **B2** | **119** | **133** | **GKAKMLSTELHNHTF** | **Wing** | **80.95%** |
|  | B3 | 141 | 156 | AECPNTNRAWNSLEVE | Wing/connector | 97.40% |
|  | B4 | 174 | 216 | RQDVSCDSKLMSAAIKDNRAVHADMGYWIESALNDTWKIEKA | β-ladder | 68.07% |
|  | B5 | 217 | 221 | FIEVK | β-ladder | 99.68% |
|  | **B6** | **223** | **244** | **CHWPKSHTLWSNGVLESEMIIP** | **β-ladder** | **98.27%** |
|  | **B7** | **250** | **268** | **PVSQHNYRPGYHTQTAGPW** | **β-ladder** | **89.29%** |
|  | B8 | 272 | 272 | R | β-ladder | 75.4% |
|  | B9 | 276 | 315 | DFDFCDGTTVVVTEDCGNRGPSLRTTTASGKLITEWCCRS | β-ladder | 25.97% |
|  | B10 | 325 | 327 | GED | β-ladder | 99.89% |
|  | B11 | 341 | 348 | KEENLVNS | β-ladder | 98.59% |
| **DENV-3** | B1 | 6 | 155 | INWKGKELKCGSGIFVTNEVHTWTEQYKFQADSPKRLATAIAGAWENGVCGIRSTTRMENLLWKQVANELNYILWENNIKLTVVVGDTIGVLEQGKRTLTPQPMELKYSWKTWGKAKIVTAETQNSSFIIDGPNTPECPSASRAWNVWEV | β-roll/wing/connector | 33.87% |
|  | B2 | 176 | 245 | TQMCDHRLMSAAVKDERAVHADMGYWIESQKNGSWKLEKASLIEVKTCTWPKSHTLWSNGVLESDMIIPK | β-ladder | 85.48% |
|  | B3 | 249 | 269 | GPISQHNYRPGYHTQTAGPWH | β-ladder | 98.39% |
|  | B4 | 271 | 272 | GK | β-ladder | 98% |
|  | **B5** | **278** | **315** | **NYCEGTTVVITENCGTRGPSLRTTTVSGKLIHEWCCRS** | **β-ladder** | **83.87%** |
|  | B6 | 322 | 332 | RYMGEDGCWYG | β-ladder | 100.00% |
|  | B7 | 336 | 349 | RPISEKEENMVKSL | β-ladder | 96.77% |
| **DENV-4** | B1 | 6 | 37 | VSWSGKELKCGSGIFIIDNVHTWTEQYKFQPE | β-roll/connector | 76.19% |
|  | B2 | 44 | 102 | SAILNAHKDGVCGIRSTTRLENVMWKQITNELNYVLWEGGHDLTVVAGDVKGVLSKGKR | Wing | 71.43% |
|  | B3 | 109 | 114 | NDLKYS | Wing | 90.48% |
|  | B4 | 124 | 148 | FTPETRNSTFLIDGPDTSECPNERR | Wing | 80.95% |
|  | B5 | 165 | 221 | TNIWMKLREGSSEVCDHRLMSAAIKDQKAVHADMGYWIESSKNQTWQIEKASLIEVK | Connector/ β-ladder | 47.62% |
|  | B6 | 223 | 243 | CLWPKTHTLWSNGVLESQMLI | β-ladder | 90.48% |
|  | B7 | 251 | 272 | FSQHNYRQGYATQTMGPWHLGK | β-ladder | 57.14% |
|  | B8 | 282 | 318 | GTTVTIQEDCDHRGPSLRTTTASGKLVTQWCCRSCTM | β-ladder | 90.48% |
|  | B9 | 330 | 330 | W | β-ladder | 100.00% |
|  | B10 | 332 | 332 | G | β-ladder | 100.00% |
|  | B11 | 337 | 348 | PLSEKEENMVKS | β-ladder | 95.24% |

**Supplementary Table S6 -** Predicted B-cell epitope coordinates and overlap with positively selected sites in Dengue virus NS1.

| **Serotype** | **B-cell epitope number** | **Epitope coordinate** | | **Overlapping positively selected sites** |
| --- | --- | --- | --- | --- |
|  |  | **Start** | **End** |  |
| **DENV-1** | **B1** | **6** | **96** | **S94N** |
|  | B2 | 109 | 121 |  |
|  | B3 | 123 | 136 |  |
|  | B4 | 142 | 154 |  |
|  | B5 | 172 | 214 |  |
|  | B6 | 218 | 221 |  |
|  | B7 | 224 | 242 |  |
|  | B8 | 254 | 256 |  |
|  | B9 | 265 | 272 |  |
|  | **B10** | **276** | **315** | **D278N, D288E/N** |
|  | **B11** | **322** | **328** | **K324R** |
|  | B12 | 342 | 348 |  |
| **DENV-2** | **B1** | **6** | **116** | **S80T,I96V, T109I/A** |
|  | **B2** | **119** | **133** | **S128L** |
|  | B3 | 141 | 156 |  |
|  | B4 | 174 | 216 |  |
|  | B5 | 217 | 221 |  |
|  | **B6** | **223** | **244** | **N234M/D** |
|  | **B7** | **250** | **268** | **Y260I, I264T** |
|  | B8 | 272 | - |  |
|  | B9 | 276 | 315 |  |
|  | B10 | 325 | 327 |  |
|  | B11 | 341 | 348 |  |
| **DENV-3** | B1 | 6 | 155 |  |
|  | B2 | 176 | 245 |  |
|  | B3 | 249 | 269 |  |
|  | B4 | 271 | 272 |  |
|  | **B5** | **278** | **315** | **I287V** |
|  | B6 | 322 | 332 |  |
|  | B7 | 336 | 349 |  |
| **DENV-4** | B1 | 6 | 37 |  |
|  | B2 | 44 | 102 |  |
|  | B3 | 109 | 114 |  |
|  | B4 | 124 | 148 |  |
|  | B5 | 165 | 221 |  |
|  | B6 | 223 | 243 |  |
|  | B7 | 251 | 272 |  |
|  | B8 | 282 | 318 |  |
|  | B9 | 330 | - |  |
|  | B10 | 332 | - |  |
|  | B11 | 337 | 348 |  |

**Supplementary Table S7 –** Estimated distance of amino acid substitutions at positively selected sites from antibody interface (Å) represented in the resolved NS1 region of 7BSC antibody complex and potential effect on antibody binding interactions as predicted by Affinity Change (ΔΔG) using mCSM-AB web server.

| **Serotype** | **Mutation** | **Distance to antibody interface (Å)** | **Predicted affinity change (ΔΔG)** |
| --- | --- | --- | --- |
| **DENV-1** | D278N | 15 | -0.78 kcal/mol (Reduced affinity) |
|  | D288E | 30 | 0.064 kcal/mol (Increased affinity) |
|  | D288N | 30 | -0.11 kcal/mol (Reduced affinity) |
|  | K324R | 11 | 0.231 kcal/mol (Increased affinity) |
| **DENV-2** | N222S | 26 | -0.279 kcal/mol (Reduced affinity) |
|  | N234M | 42 | -0.013 kcal/mol (Reduced affinity) |
|  | N234D | 42 | 0.035 kcal/mol (Increased affinity) |
|  | Y260I | 29 | -0.091 kcal/mol (Reduced affinity) |
|  | I264T | 23 | 0.085 kcal/mol (Increased affinity) |
| **DENV-3** | I287V | 15 | -0.381 kcal/mol (Reduced affinity) |

Predicted Affinity Change (ΔΔG) values < |1| kcal/mol represent weak predicted effects.

**Supplementary references**

[1]	D. R. Perera, N. D. Ranadeva, K. Sirisena, and K. J. Wijesinghe, “Roles of NS1 Protein in Flavivirus Pathogenesis,” *ACS Infect. Dis.*, vol. 10, no. 1, pp. 20–56, Jan. 2024, doi: 10.1021/acsinfecdis.3c00566.

[2]	N. T. N. Lo, S. Z. Roodsari, N. L. Tin, M. P. Wong, S. B. Biering, and E. Harris, “Molecular Determinants of Tissue Specificity of Flavivirus Nonstructural Protein 1 Interaction with Endothelial Cells,” *J. Virol.*, vol. 96, no. 19, pp. e00661-22, Sep. 2022, doi: 10.1128/jvi.00661-22.

[3]	R. Kraivong, S. Hattakam, S. Thiemmeca, and P. Avirutnan, “The Dengue NS1 epitope landscape: a blueprint for next-generation vaccines and therapeutics,” *Curr. Opin. Virol.*, vol. 73, p. 101488, Dec. 2025, doi: 10.1016/j.coviro.2025.101488.

[4]	Y.-C. Lai *et al.*, “Antibodies Against Modified NS1 Wing Domain Peptide Protect Against Dengue Virus Infection,” *Sci. Rep.*, vol. 7, no. 1, p. 6975, Aug. 2017, doi: 10.1038/s41598-017-07308-3.

[5]	S.-W. Wan *et al.*, “Therapeutic Effects of Monoclonal Antibody against Dengue Virus NS1 in a STAT1 Knockout Mouse Model of Dengue Infection,” *J. Immunol.*, vol. 199, no. 8, pp. 2834–2844, Oct. 2017, doi: 10.4049/jimmunol.1601523.

[6]	R. Kraivong *et al.*, “Cross-reactive antibodies targeting surface-exposed non-structural protein 1 (NS1) of dengue virus-infected cells recognize epitopes on the spaghetti loop of the β-ladder domain,” *PLOS ONE*, vol. 17, no. 5, p. e0266136, May 2022, doi: 10.1371/journal.pone.0266136.

[7]	A. K. I. Falconar, P. R. Young, and M. A. Miles, “Precise location of sequential dengue virus subcomplex and complex B cell epitopes on the nonstructural-1 glycoprotein,” *Arch. Virol.*, vol. 137, no. 3, pp. 315–326, Sep. 1994, doi: 10.1007/BF01309478.

[8]	M. L. Jones *et al.*, “Computational Identification of Antibody Epitopes on the Dengue Virus NS1 Protein,” *Molecules*, vol. 22, no. 4, Apr. 2017, doi: 10.3390/molecules22040607.

[9]	P. K. Nagar, D. Savargaonkar, and A. R. Anvikar, “Detection of Dengue Virus-Specific IgM and IgG Antibodies through Peptide Sequences of Envelope and NS1 Proteins for Serological Identification,” *J. Immunol. Res.*, vol. 2020, p. 1820325, 2020, doi: 10.1155/2020/1820325.

[10]	L. Jiang, J.-M. Zhou, Y. Yin, D.-Y. Fang, Y.-X. Tang, and L.-F. Jiang, “Selection and identification of B-cell epitope on NS1 protein of dengue virus type 2,” *Virus Res.*, vol. 150, no. 1, pp. 49–55, Jun. 2010, doi: 10.1016/j.virusres.2010.02.012.

[11]	N. L. Warner *et al.*, “Expansion and Refinement of Deep Sequence-Coupled Biopanning Technology for Epitope-Specific Antibody Responses in Human Serum,” *Viruses*, vol. 12, no. 10, Sep. 2020, doi: 10.3390/v12101114.

[12]	L. B. Rocha *et al.*, “Epitope Sequences in Dengue Virus NS1 Protein Identified by Monoclonal Antibodies,” *Antibodies*, vol. 6, no. 4, Oct. 2017, doi: 10.3390/antib6040014.

[13]	“Dengue Situation in India, NCVBDC,” Dengue Situation in India, NCVBDC. Accessed: Sep. 17, 2026. [Online]. Available: https://ncvbdc.mohfw.gov.in/index1.php?lang=1&level=1&sublinkid=5776&lid=3690

[14]	K. Katoh and D. M. Standley, “MAFFT Multiple Sequence Alignment Software Version 7: Improvements in Performance and Usability,” *Mol. Biol. Evol.*, vol. 30, no. 4, pp. 772–780, Apr. 2013, doi: 10.1093/molbev/mst010.

[15]	D. P. Martin *et al.*, “RDP5: a computer program for analyzing recombination in, and removing signals of recombination from, nucleotide sequence datasets,” *Virus Evol.*, vol. 7, no. 1, p. veaa087, Jan. 2021, doi: 10.1093/ve/veaa087.

[16]	S. L. Kosakovsky Pond *et al.*, “HyPhy 2.5—A Customizable Platform for Evolutionary Hypothesis Testing Using Phylogenies,” *Mol. Biol. Evol.*, vol. 37, no. 1, pp. 295–299, Aug. 2019, doi: 10.1093/molbev/msz197.

[17]	M. C. Jespersen, B. Peters, M. Nielsen, and P. Marcatili, “BepiPred-2.0: improving sequence-based B-cell epitope prediction using conformational epitopes,” *Nucleic Acids Res.*, vol. 45, no. Web Server issue, pp. W24–W29, Jul. 2017, doi: 10.1093/nar/gkx346.

[18]	H.-H. Bui, J. Sidney, W. Li, N. Fusseder, and A. Sette, “Development of an epitope conservancy analysis tool to facilitate the design of epitope-based diagnostics and vaccines,” *BMC Bioinformatics*, vol. 8, p. 361, Sep. 2007, doi: 10.1186/1471-2105-8-361.

[19]	D. E. V. Pires and D. B. Ascher, “mCSM-AB: a web server for predicting antibody–antigen affinity changes upon mutation with graph-based signatures,” *Nucleic Acids Res.*, vol. 44, no. Web Server issue, pp. W469–W473, Jul. 2016, doi: 10.1093/nar/gkw458.

[20]	“Support | pymol.org.” Accessed: Jan. 18, 2026. [Online]. Available: https://www.pymol.org/support.html
